# Health behavioural change – the influence of social-ecological factors and health identity

**DOI:** 10.1080/17482631.2025.2458309

**Published:** 2025-01-31

**Authors:** Malin Eriksson, Linda R Sundberg, Ailiana Santosa, Helena Lindgren, Nawi Ng, Kristina Lindvall

**Affiliations:** a Department of Social Work, Umeå University, Umeå, Sweden; b Department of Psychology, Umeå University, Umeå, Sweden; c School of Public Health and Community Medicine, Sahlgrenska Academy, University of Gothenburg, Gothenburg, Sweden; d Department of Computing Science, Department of Community Medicine and Rehabilitation, Umeå University, Umeå, Sweden; e Department of Epidemiology and Global Health, Umeå University, Umeå, Sweden

**Keywords:** Health behaviour, social-ecological, health promotion, health interventions, health identity, grounded theory

## Abstract

Health behaviour is crucial for influencing health, making it a key component in health promotion. However, changing behaviours is complex, as many factors interact to determine health behaviours. Information, awareness, and knowledge are important but not enough. It is essential to move beyond focusing solely on individual psychological and cognitive factors to an understanding of the complex processes involved in health behaviour change. Social-ecological models account for these complex processes but risk being overly broad and all-encompassing. This qualitative grounded theory study explores how individual, interpersonal, and environmental factors interplay to influence health behaviour, and examines how social-ecological models in health promotion can be tailored to address different ecological needs. Participants were recruited from a community-based cardiovascular disease-prevention program in Northern Sweden. Data was collected through in-depth interviews about health and health behaviours throughout the life course among middle-aged men and women. The results illustrate how factors obstructing or enabling health behaviours vary in patterned ways for individuals with different health identities. Social-ecological interventions could be more effective if adapted to the specific needs of people with different health identities. In addition to screening for various risk factors, screening for health identities could be helpful in designing social-ecological health-promoting interventions.

## 1. Introduction

### 1.1. Health behaviours as health determinants

Our health is influenced by our behaviours, particularly regarding physical activity, eating habits, smoking and substance use, and healthcare seeking and adherence to treatment and prevention (e.g., vaccination). Consequently, health behaviour plays a central role in explaining and influencing health and health inequalities and is, therefore, a key component in public health interventions and health promotion (Clark, 2002). Health behaviours are “…overt behavioural patterns, actions and habits that relate to health maintenance, to health restoration and to health improvement” (Gochman, 1997, p. 3). A broad range of behaviours fall into that definition: not only behaviours directly impacting health (e.g., physical activity) but also our social practices, such as civic engagement and involvement in social networks. While the relationship between some behaviours and specific health outcomes is clear (e.g., smoking and lung cancer), the mechanisms linking various behaviours to other health outcomes for different groups of people remain complex (Conner & Norman, 2017). Social media usage could, for example, act as both a trigger and a barrier for healthy behaviours such as physical activity or a healthy diet, and these patterns may vary among different population groups.

Interventions to influence health behaviour are essential, although not easy, as many complex factors interact in determining health-related behaviours. Information, awareness and knowledge are key, yet insufficient. Most people continue with unhealthy behaviours (e.g., smoking or eating junk food) despite being aware that this behaviour is unhealthy. Changed attitudes or increased knowledge do not continually transform into action (Grabowski, 2015). While some key determinants might be the same for numerous health behaviours, many are unique to certain behaviours (Conner & Norman, 2017). Hence, there is a need to move beyond a sole focus on individual psychological and cognitive factors to understand and influence health behaviours and achieve a comprehensive understanding of the complex processes involved in behavioural change.

Different theoretical models for health behaviour can guide health interventions and health promotion programmes. Several of these are based on health psychology and mainly target individuals, such as the Health Belief Model (Becker, 1974; Janz & Becker, 1984), the Theory of Reasoned Action (Ajzen, 1985), the Theory of Planned Behaviour (Ajzen, 1991) and the Trans-theoretical Model and Stages of Change (DiClemente & Prochaska, 1998). Other models highlight interpersonal relations and group-based interventions, such as Social Cognitive Theory (Bandura, 1986, 1998) and Social Networks and Social Support Models (Heany & Israel, 2002). Community Organisation and Community Building Models (Minkler & Wallerstein, 2002), Diffusion of Innovations (Rogers, 2003) and Communication Theories (Simons-Morton et al., 2012) instead focus on communities, schools or worksites as the primary intervention setting for influencing health behaviours.

### 1.2. Social-ecological approaches to health behavioural change

Social-ecological models in health promotion emphasize that individuals are embedded within multiple social systems and acknowledge the interactive characteristics of individuals and environments that influence health outcomes (Golden & Earp, 2012; Stokols, 1996). Therefore, social-ecological models aim to focus simultaneously on multiple factors and their interactions in different ecological systems (individual, interpersonal and environmental) to understand and influence health behaviours (Stokols, 1996). This could be done, for example, by simultaneously trying to increase individuals’ health literacy and change community norms (Schölmerich & Kawachi, 2016). McLeroy et al. (1988) identified five levels of influence on health-related behaviours and conditions: (1) intrapersonal or individual factors, (2) interpersonal factors, (3) institutional or organizational factors, (4) community factors and (5) public policy factors. Furthermore, social-ecological models acknowledge interactions between and the interdependence of factors within and across all ecological levels in understanding and influencing health-related behaviours. Behaviour is affected by multiple levels of influence, and individual behaviour shapes and is shaped by the social environment (McLeroy et al., 1988). Based on these assumptions, appropriate changes in the social environment will lead to changes in individuals, while supporting individuals in the population will contribute to environmental changes.

Social-ecological models are clearly located within the broader social determinants of health framework. The World Health Organization (WHO) defines social determinants of health (SDH) as “the conditions in which people are born, grow, live, work and age, including the health system” (WHO Commission on the Social Determinants of Health CSDH, 2008). The SDH are commonly illustrated by the Dahlgren and Whiteheads model, which shows how biological and individual lifestyle factors determine health; in turn, these are determined by social and community networks, living and working conditions, and the overall socioeconomic, cultural and environmental conditions in society (G. Dahlgren & Whitehead, 1991). The conceptual framework of the SDH, outlined by the WHO, stresses that unequal access to power, resources and prestige in society influences the exposure, vulnerability and effect of various material, psychosocial and behavioural circumstances that determine health and health behaviours. A low socioeconomic position, for example, limits the ability to manage the costs required for a healthy lifestyle (e.g., exercise equipment, healthy food, transportation costs). Social position in a society may also influence health-related behaviours through knowledge and health literacy (i.e., the ability to acquire, understand, value and use information to maintain and promote health) but also via social norms and habitus that differ between socioeconomic groups. As such, unhealthy behaviour such as smoking or overeating can be viewed as a means of managing difficult life circumstances because of low socioeconomic position (Bartley, 2004). In addition, a low socioeconomic position increases exposure and vulnerability to long-term stress, such as economic strain, social exclusion or unemployment, which may further limit the ability to maintain a healthy lifestyle (Bartley, 2004). The SDH focuses on “the causes of the causes” — i.e. what factors causing health-related behaviours . Understanding these root causes for different groups and individuals could be critical for guiding health interventions and health promotion.

The premise of social-ecological models for understanding and influencing health behaviour is that they embrace the complex processes and SDH involved in behavioural change. However, embracing and addressing these complex processes is a complex task. Practical examples of social-ecological health promotion interventions are still scarce, and the gap between theory and practice is wide (Golden & Earp, 2012; Schölmerich & Kawachi, 2016). Golden and Earp (2012) reviewed 157 articles reporting on health-promotion programmes. They concluded that most (95%) of the programmes contained individual-level activities, about 65% included interpersonal activities, while only 20% of the programmes contained community- and policy-level activities. In addition, fewer than 10% of the included articles identified an ecological theoretical frame as a basis for the interventions (Golden & Earp, 2012). There is, therefore, little guidance on how to design social-ecological health-promotion interventions.

Moreover, there is a lack of evidence that multilevel interventions significantly impact health outcomes more than single-level interventions (Schölmerich & Kawachi, 2016). Another challenge with social-ecological interventions is that they risk being too inclusive and all-encompassing by trying to address all possible health-related variables at different levels since everything is reciprocally connected to everything else (Wold & Mittelmark, 2018). Social-ecological interventions might try to fit everyone and thereby risk fitting no one. Stokols (1996), pp. 287–288) emphasizes that “Overly inclusive models are not likely to assist researchers in targeting selected variables for study, or clinicians and policymakers in determining where, when, and how to intervene”. Also, the same environmental conditions that an intervention aims to influence (e.g., community norms) might affect people’s health very differently based on personal factors such as personality, attitudes and individual resources (Stokols, 1996). Therefore, intervention strategies based on social-ecological models in health promotion might need to be tailor-made for each behaviour and population (Elder et al., 2007). It is thus crucial to carefully investigate the complex interactions between different ecological systems for different groups and individuals to understand for whom a specific intervention might work. By so doing, social-ecological interventions can become tailor-made to fit the specific ecological patterns and needs of various subgroups, thereby ensuring a “fit” between the individual and their environment.

## 2. Aim

This study explores the interplay of individual, interpersonal and environmental factors in influencing health-related behavioural changes among middle-aged individuals in Northern Sweden. It also discusses how social-ecological models in health-promotion interventions can be tailored to address different ecological patterns and needs.

## 3. Methods

### 3.1. Study setting—the Västerbotten intervention programme

The participants in this qualitative study were recruited from a community-based cardiovascular disease-prevention programme called the Västerbotten Intervention Programme (VIP) in Västerbotten county in Northern Sweden. The VIP was launched in one municipality in 1985 and, after that, successively implemented across the county; since 1995, it has been integrated into ordinary primary-care routines in the county. The integration implies that all middle-aged persons, upon reaching the ages 40, 50 and 60, are invited to participate in systematic cardiovascular diseases (CVD) risk factor screening and examination, a comprehensive health survey including lifestyle habits and socioeconomic and psychosocial factors, and an individual health dialogue with a specialized nurse (Norberg et al., 2010). During these health dialogues, participants receive feedback on their survey and measurements. The participant’s risk profile is visualized as a star, where blunt tips indicate greater risk and low risk is illustrated as a star with sharp tips. The use of the VIP star aims at facilitating participants’ understanding of the associations between lifestyle, behaviours and CVD risk factors. The overarching goal of the VIP is to motivate participants to maintain healthy habits and to support and motivate those with multiple risk factors to modify their behaviours (Lindholm et al., 2018).

### 3.2. Study design

This study was designed as a qualitative, social constructionist grounded theory (GT) study (Charmaz, 2006). Data was based on in-depth interviews about health and health behaviours over the life course among middle-aged men and women in Northern Sweden. In line with the basic principles of social constructionist GT, we used an abductive approach in which data collection and analysis were conducted in parallel. Consequently, the results from the first interview guided the development of the final interview guide and, thereby, the subsequent interviews.

### 3.3. Sampling and study participants

All participants in this study had participated in the VIP during 2018–2019, immediately preceding the interview and could thus recall their CVD risk profile as identified in the programme. We aimed to invite participants with a variation in their sociodemographic and socioeconomic profiles—that is, their areas of residence (urban and rural), educational length (shorter and longer), sex (men and women) and age group (40, 50 or 60, respectively). Following ethical approval, we obtained a list with a random sample of prior VIP participants who fulfilled the inclusion criteria (as specified above) from the Norr Register Centre (Registercentrum Norr, 2023), which is responsible for the VIP register. Receiving contact information from the register implied that no gatekeepers involved in the intervention program (e.g., primary health care nurses) were needed in order to reach out to potential participants. As a first step, we sent an information letter about the study via ordinary mail. Approximately one week after the letter was sent, the responsible researchers (ME, LS, KL) called the potential participants to provide oral information about the study and to extend a personal invitation. Eighty-six individuals received the information letter, of which 69 declined participation or could not be reached by phone after three attempts. Those who declined participation mentioned time constraints and lack of interest.

In total, 17 individuals (13 women and four men) agreed to participate. The sample had variations in age, education and residence in rural and urban areas of Västerbotten, as illustrated in Table 1.

**Table 1.** Sociodemographic description of the participants.

Sex	Age			Geographical residence		Educational background	
	40	50	60	Urban	Rural	Short	Long
Women (*N*=13)	7	5	1	4	9	3	10
Men (*N*=4)		1	3	1	3	2	2

### 3.4. Data collection

Semi-structured interviews were conducted in Swedish between the spring and early fall of 2021. The interviews were preceded by a pilot interview in the spring of 2020 to appraise how the interview guide worked. The pilot interview led to only minor changes in the guide. Due to the valuable information obtained, this pilot interview was included in the final dataset (i.e., as one of the 17 participants).

A thematic interview guide with open questions was constructed and used during the interviews. We wanted to capture participants subjective views on health and health behavioural change over their life course. Therefore, each interview started with an opening question on how the participant viewed and defined health and what they included when thinking about their health. After that, the participants were asked to describe, on an illustrated timeline, how their health had fluctuated over their lifespan. Each participant decided on the line’s starting point (in time). Some participants started describing their “health journey” from their childhood, while others preferred to reflect upon their health during recent years. Following this, the participants were asked to describe what factors contributed to times of good versus poorer health on their health line. Steered by the overall aim of the project, and by our theoretical pre-understanding of the social determinants of health, we specifically probed their behaviour and lifestyle, social situation, social networks, work situation, significant life events and experience with COVID-19. One final question included the role of VIP in health behaviour change. Issues related to COVID-19 were judged to require a separate analysis and are therefore not utilized in this study. Likewise, the role of VIP is not analysed in the current study.

Due to the ongoing COVID-19 pandemic, all informants but one chosed to be interviewed via telephone. The final informant preferred to be interviewed via video to be able to see the researcher. The interviews lasted from 40 to 90 minutes. After each interview, memos were written and later used in discussions between the co-authors and in the preliminary analysis. All interviews were tape-recorded and transcribed verbatim to prepare for the coding process.

### 3.5. Analysis

We conducted a social constructivist GT analysis, in line with Charmaz (2006) and Clarke (2005). This approach involved following the basic coding procedures in GT, with initial, focused and theoretical coding (Charmaz, 2006). In line with the principle of constant comparisons, the coding procedure was not a linear process but rather an iterative process with constant comparisons between data, initial and focus codes to make analytical sense of the data and help conceptualizing our ideas (Charmaz, 2006). Social constructionist GT proposes abduction and grounded theorizing, rather than induction and the construction of theory (Clarke, 2005). Thus, we used the social-ecological model as a sensitizing concept, where the theoretical model suggested directions along which to look in the data without steering precisely what to see (Clarke, 2005).

**Initial coding** was done on all interviews, line by line, without having specific theoretical ideas in mind. The three main authors (ME, LS, KL) were responsible for the initial coding of the interviews they had, respectively, conducted. They also performed a first sorting of their initial codes to facilitate a discussion of what was present in the data. Next, they met to discuss and negotiate their sorting of initial codes. Based on these discussions, two separate analytical paths were decided on for the subsequent analyses.

This article is based on the rich subset of initial codes containing information about various factors perceived and described as influencing health behaviour changes. These were sorted into clusters due to their content. Some clusters made particular analytical sense for the aim of this study, i.e., clusters of codes referring to s*elf-image, motives* and *driving forces, social organization of daily life, social networks, living environment, working environment, and nature*. Another relevant cluster of codes referred to the influence on health behaviour of an overall *health identity* – that is,

“*observations and expectations concerning their health, their knowledge about health and in what ways their health is related and comparable to the health of others*” (Grabowski, 2015, p. 141).

The **focused coding** involved sorting, comparing and synthesizing codes within and between these clusters of initial codes. This process revealed an overlap between codes related to health identity and all other clusters of codes. This overlap was utilized to construct categories and sub-categories to conceptualize “ideal types” of health identities grounded in our data. Creating ideal types is “an attempt to create order out of seemingly heterogeneous events by accentuating homogeneous attributes” (Hendricks & Breckinridge, 1973, p. 32). These ideal types thus captured some essential features of health identity in relation to health behaviour change (Ritzer, 2000, p. 115). Following Weber (1903/1949, quoted in Ritzer, 2000, p. 115), the ideal types were viewed as analytical constructs (i.e., not found in their “purity” in our data such that each participant represented solely one ideal type) but were still generated from our empirical data.

**Figure 1.**
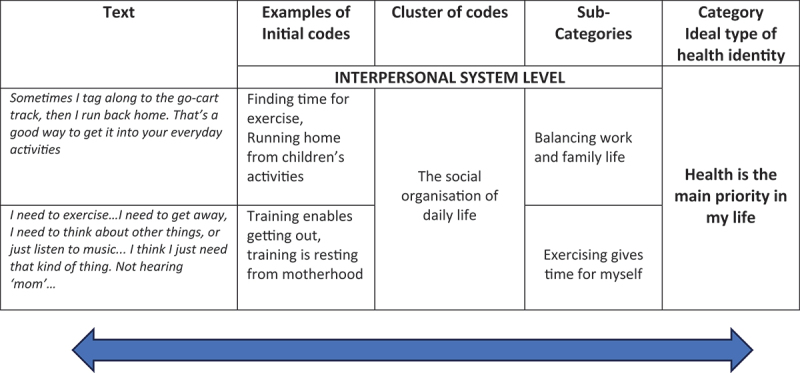
Illustration of the coding process.

Finally, in the **theoretical coding**, we linked our categories of ideal types and their assigned sub-categories and codes to our sensitizing concept of the social-ecological model. By that, groups of codes assigned to each identified ideal type could be sorted into different levels of the social-ecological model. Finally, the results were illustrated by constructing a positional map of “the major positions taken in the data” (Clarke, 2005, p. 126) about factors in different ecological systems perceived as obstructing or enabling health behaviour change for various ideal types of health identities. Figure 1 illustrates the coding process of moving back and forth between text, codes and categories.

### 3.6. Ethical considerations

The Swedish Ethical Review Authority (dnr: 2019–02924 and dnr: 2020–02985) approved the study. All participants received oral and written information about the study before deciding on their participation. Verbal informed consent was completed before the interview started. During the interviews, we reminded the participants that their participation was voluntary and that they could withdraw at any time. We also emphasized that the participants could decline to answer some questions if they felt uncomfortable answering. The interviews were conducted as an informal conversation in which the participants decided what they wanted to share concerning their health and health behaviour change. The fact that most interviews were conducted by phone meant that the interviewer did not see most participants. Some of the participants referred to themselves as “being fat” – a description that the researcher could not confirm. This could be viewed as a limitation, as it could decrease the trustworthiness of the data. However, we also sensed that not being seen (and thereby evaluated) by the researcher allowed the participants to “speak their heart” even more. This could be particularly important when sharing sensitive information about difficult life experiences and illness.

The potential risk of researchers influencing participants on their willingness to contribute to research is an important ethical issue. One of the authors [KL] is involved in the strategic planning of the VIP intervention, but none of the researchers are involved in the implementation of the program. Thus, the researchers had not met study participants in any other role previously (e.g., as patients or clients). To further avoid the potential risk of influencing participation, we adjusted whom each responsible researcher invited from the list of names obtained from Norr Register Centre. We did not contact anyone whose name was known to us by any means.

## 4. Results

### 4.1. Three ideal types of health identity and their perceptions of health behaviours

The GT analysis resulted in the construction of three overall categories describing different ideal types of health identities—that is, homogeneous attributes regarding expectations and knowledge about health and views of their health compared with that of others. The analysis revealed that these ideal types shared perceptions about obstructing and enabling factors for health behaviour change emanating from different ecological levels. Table 2 gives an overview of codes, clusters of codes and sub-categories that were utilized to construct categories describing three ideal types of health identities.

**Table 2.** Categories of three ideal types of health identity with their assigned codes, constructed from the social constructivist grounded theory (GT) analysis.

ECOLOGICAL SYSTEM LEVEL	CLUSTERS OF CODES	CATEGORIES		
		IDEAL TYPE 1 Health is the main priority in my life	IDEAL TYPE 2 Life stands in the way of my health	IDEAL TYPE 3 Health is not a major concern in my life
		SUB-CATEGORIES		
INDIVIDUAL SYSTEM LEVEL	*Self-image*	- I look at the bright side of life - I conquer setbacks - I am determined to live a healthy life	- I put others before myself - I do not have many options	- I prefer to be on my own - I’m fine, after all
	*Motivation and driving forces*	- I set health goals for myself - Exercising is an enjoyable must - Living healthy to reduce future illness	- I want to change, but I’m trapped in life - Life stands in the way of health - A healthy life is but a dream	- I would rather invest in health tomorrow … - Previous attempts have failed - Sustainable lifestyle change is almost impossible
INTER- PERSONAL SYSTEM LEVEL	*The social organisation of daily life*	- Balancing work and family life - Adjusting exercise after life circumstances - Exercising gives time for myself	- Domestic life obstructs a healthy life - My daily life is full of stress	- Eating healthy is out of my control - Irregular working hours obstruct healthy food
	*Social networks*	- Family support is crucial - My training buddy supports change - Close friends are important for health	- Online support compensates for a lack of offline support - No time for socialising - Ordinary people cannot understand my life	- Close family is enough - My way of living obstructs socialising - I refuse to exercise with people around
ENVIRON- MENTAL SYSTEM LEVEL	*Living environment*	- Neighbourhood relations mean safety - Proximity facilities socialising - Proximity to work enables walking or biking	- The home environment is demanding - Neighbourhood support is important - Proximity to school and work eases everyday life	- My home is my castle - Living in peace and calm is important - Moving between environments obstructs physical exercise
	*Working environment*	- Colleagues mean a lot - Enjoying work gives energy for life and health - Problems at work are negative for health	- Worn out from work - Too tired after work for an active life - No other work opportunities for me	- I prefer not to interfere with colleagues - Outdoor job is good for health - Dissatisfaction at work is bad for health
	*Nature*	- Nature is health-promoting - Nature gives recovery - Nature gives a sense of freedom	- Nature gives strength and recovery - The forest is healing	- Nature gives peace and calm - Nature facilitates physical activity

Ideal type 1 was labelled *Health is the main priority in my life*. This was our data’s most common health identity because many codes referred to this ideal type. Ideal types 2 and 3 were less common, in the sense that we had fewer codes in our data that refereed to these health identities. Still, these ideal types are visible and grounded in our data. We labelled ideal type 2 as *Life stands in the way of my health* and ideal type 3 as *Health is not a major concern in my life*. While ideal type 1 described few barriers to health behavioural change, ideal types 2 and 3 expressed many obstacles. Despite the differences between these ideal types, they also shared similarities. All ideal types had an inclusive view of health. Overall, health was viewed as the ability to “manage life” satisfactorily. In addition, all three ideal types shared perceptions that everyone is responsible for one’s health and that being healthy requires personal investment. There were also differences in how they viewed their health, their knowledge about health and how they viewed their health compared with that of others, implying different health identities.

Given that our constructed ideal types are analytical constructs (albeit grounded in the data), the same individual could exhibit character traits from several ideal types. Still, we believe most individuals may have a dominant health identity with assigned characteristics, as found in the results. Below, we account in detail for the different ideal types and discuss how factors in different ecological systems are perceived as influencing health behavioural change. We use the pronoun they throughout the text below to facilitate the reading regarding each ideal type.

#### 4.1.1. Ideal type 1: health is the main priority in my life

This ideal type describes themselves as being devoted to living a healthy life. Their self-image is that they are curious and determined to reach their (health) goals. Therefore, they enjoy setting goals and constantly challenging themself. Setbacks are seen as part of the process, but they are determined to conquer setbacks by staying positive and focusing on possibilities rather than obstacles in life. This is mirrored in their motivation and driving forces since, for example, physical exercise has become a daily routine and a part of everyday activities. Physical activity is seen as an enjoyable must and thus prioritized despite many daily chores and demands. Even if there is no time to go to the gym, they find time to, for example, take a short walk during lunchtime or go for a jog while taking the kids to their leisure activities: “*Sometimes I tag along to the go-cart track, then I run back home. That’s a good way to get it into your everyday activities*” (woman, 40 years old, participant 2). These priorities are based on conscious choices and knowledge; they know they need physical exercise for their mental health and weight. Further, previous personal and family history of diseases has made them aware that investing in health today is important to reduce the risk of future illness. One of the participants who had experienced previous burnout and chronic pain commented,

*I think I’ve learned how to handle it better today … when I look back, I can see that I was a bit tougher on myself … today, I do understand more regarding balancing … if you’re too tense, you shouldn’t work out; then you just get more tense* … .

(Woman, 50 years old, participant 7)

Regarding social networks, this ideal type values relations with family and friends and views some of them as important role models in life. Family is the most important social network since family members are always there for each other. Having close people to talk to is important, and social support is seen as essential for health. According to this ideal type, close people can support health behavioural change by providing emotional and instrumental support and giving ultimatums for a change when needed. Thus, this ideal type nurtures friendship based on trust, reciprocal help, and support. In contrast, however, some informants that mainly aligned with this ideal type also stated that they had consciously withdrawn from social relations that were draining, too demanding or made them feel bad:

*Yes, but it’s important to have friends and a social network that give you strength, too, and not just eat energy. So, I have chosen to … with years, I feel that it’s just hard work, then I drop out of it, and it fades away*.

(Man, 60 years old, participant 17))

Regarding physical activities, this ideal type describes how they combine socializing with physical activities, such as working out or taking walks with friends. Having a “training buddy” is also common for this ideal type. Several participants expressed the value of setting training goals with friends, such as signing up for a marathon or losing weight. These activities added competitive and supportive components that further spurred them on and helped them reach health goals. In addition, going out with a friend to exercise was seen as enabling “quality time on one’s own” and a pause from a stressful and often demanding family life:

*I have a friend who is also very interested in exercising, and we have challenged each other in going to various trails, et cetera. We have gone to … trails, yes, but various competitions we’ve gone to. The High Coast Trail, yes, we’ve been all over the place. We have trained together to achieve it, and really it’s, like, to get a chance to get away from things, from it all. So, it’s been fun*.

(Woman, 40 years old, participant 2)

This ideal type also views living and working environments as important for health and health behaviour. Living close to friends and family facilitates socializing. In addition, good relations with neighbours are seen as an important source of help and support when needed. Furthermore, proximity to nature is viewed as essential. Spending time in nature promotes physical activity, gives a sense of freedom and enables recovery from stress. Several participants also highlighted how having a dog facilitates outdoor physical activities. In addition, proximity to the workplace was valued since it enables walking or biking to work. A good working environment was also seen as important for health, while problems at work affected health negatively. Some also expressed that their position, for example, as a health professional, required a healthy lifestyle to be viewed as credible. Having good colleagues can fill social needs, and feeling valued at work was perceived as important. In addition, having a job that one enjoys gives energy for life and health in general. In sum, this ideal type has high expectations of health, is knowledgeable about what is required for health and generally rates their health as better in comparison with that of others.

#### 4.1.2. Ideal type 2 – life stands in the way of my health

This ideal type expresses a need for health behavioural change but sees no possibility of doing so due to a challenging life situation, which stands in the way of any health behavioural changes. Their self-image is that they have few options in life and that they put others before themselves, which is why they cannot prioritize their health: “… *my health has always come second*” (woman, 40 years old, participant 11). Thus, investing in one’s health, such as eating healthy or becoming physically active, would be possible only if challenges in life could be solved. The current job, health or family situations take all energy and leave no room for (health behavioural) changes. Even if they wish to become active for health reasons, their health cannot be prioritized in their demanding daily life:

“*Unfortunately you’re so worn out at the end of a workday that you don’t have energy for anything else, and when you have [my disease], then it’s extra hard, so to speak* … ”(man, 60 years old, participant 12).

Among the participants representing this ideal type, it was common to have family members—not least children—with various health problems. This fact overshadowed their whole life situation and affected their daily life. The organization of daily life was described as stressful and demanding, and the daily routines around food, sleep, school and work took a lot of energy to manage. This daily struggle to get through the day gave them no time left to invest in their health. Hence, this ideal type expresses an overall lack of control over their life and health.

For this ideal type, the daily struggle to manage life obstructs socializing with friends and family. Several participants explained how their social networks had decreased or even vanished due to their demanding life situation. On the other hand, representatives of this ideal type also commented that “ordinary people” cannot understand their situation, so socializing with others was not particularly appealing. In contrast, socializing online was perceived as valuable. Through the Internet, this ideal type gains access to important social support groups and social media channels in a more accessible and less demanding way. These online social networks are available 24/7 – whenever they can log in. The participants representing this ideal type explained how the social support they lacked offline was available online. Sharing with people who have similar experiences of struggles in life was viewed as a significant source of help and support. Likewise, participating in online chat rooms or platforms was described as giving access to valuable information and advice on handling challenging life situations, such as those concerning their children.

*There isn’t really a real human being, a physical person that you meet … there are various forums and the like online, you do that – otherwise, you wouldn’t survive – and to exchange ideas and try to find solutions and such. You try to find solutions on your own, and it’s actually great with the Internet, various forums, et cetera*.

(Woman, 40 years old, participant 3)

Thus, socializing online is perceived as compensating for the lack of societal support and supportive offline social networks.

Regarding environmental factors, this ideal type—like ideal type 1 – values proximity to school and work since it eases the organization of everyday life. When available, support from neighbours is seen as important. Participants representing this ideal type generally have occupations in which heavy manual work is typical and, even if wished for, changing profession is not seen as an option. Heavy physical work is perceived as wearing on their health.

Despite low energy and little time for leisure, this ideal type acknowledges the significance of being outdoors in nature. Whenever possible, walks in the forest are perceived as reducing stress and giving strength and recovery, which they wish to get more of in the future.

*I know that, during periods when I’ve been thinking a lot, it’s been a help many times to go for a walk. I walked a lot one winter here when I was under a lot of stress and pressure up on the mountain*.

(Woman, 50 years old, participant 15)

In conclusion, this ideal type has low expectations of their health behaviour; they know what is required for health but see no opportunities to act upon this awareness. Consequently, they rate their health as poorer than that of others.

#### 4.1.3. Ideal type 3 – health is not a major concern in my life

Ideal type 3 sees no significant need for health-related behavioural change since health is not a major concern in their life. They describe themselves as very independent and not too concerned about others’ opinions: “They admit that they do not have the healthiest lifestyle since they are overweight, for example, and physically inactive; however, overall, they are still doing fine.

*My health has been rather steady, sort of. I have neither had any large issues with my health, really, rather acceptable test values in relation to … how I look, so to speak … I do have a BMI of 43–44, something, and that is really terribly high. I do know that* … .

(Woman, 50 years old, participant 13)

This ideal type expresses low motivation for health behaviour changes, even though they express that it would probably be good for them: “*But it is, I don’t feel bad, I’m not in pain, nothing like that, so I simply have the wrong motivation” (*woman, 40 years old, participant 4). Therefore, they tend to postpone any plans for physical exercise or weight loss and would prefer to do it some other time. The fact that they generally feel well despite being overweight or physically inactive make postponing any health behavioural change easier. In addition, this ideal type is generally sceptical about the possibility of achieving sustainable lifestyle changes. Some participants who mainly represented this ideal type said they had occasionally tried various diets and exercises. Still, none worked over time, decreasing their motivation for health behavioural change.

The independent lifestyle spills over into the social situation of this ideal type. This person claims to have relatively limited social needs; thus, their social network is rather small: “*I guess I am a big … lone wolf, so to speak. I don’t barge in on people, or, I am by myself, that’s how I work*” (man, 60 years old, participant 12). This need to be on their own might obstruct health behaviour change. One of the participants who contributed to the construction of this ideal type described how they refused to exercise with other people around and, therefore, never went to a gym. In some interviews, it became clear that participants representing this ideal type had previous negative experiences with social networks, and therefore tended to withdraw from other people. They better fend for themselves rather than expect help from others. Furthermore, irregular working hours and a preference to be alone influenced their meal habits. This ideal type tends to eat what is readily available at irregular mealtimes, which does not easily result in making the healthiest choices.

Regarding environmental factors, this ideal type considers living in peace and calm is important for their health. Being at home with no disturbances facilitates recovery from work and stress. Their home is perceived as a haven, and they prefer not to have too many people around in this peaceful environment: “ … *being left alone, not having to be around people all the time. That’s really important to me” (*woman, 40 years, participant 4). Likewise, moving between different living environments is perceived as something that disturbs their routines related to physical activities or food habits. This ideal type states that it is easier to go for a walk or jog in an environment known to them while doing so is almost impossible in an unknown environment. Concerning their working environment, they prefer to work alone without interfering too much with colleagues. However, like the other ideal types, this person enjoys being out in nature and the peace and calm that nature gives. In sum, this ideal type has no specific expectations for their health behaviour; they state that they are not too concerned about health and thus avoids comparing their health with that of others.

### 4.2. The ecological system of obstructing and enabling factors for health-enabling behaviour among different ideal types of health identity

**Figure 2.**
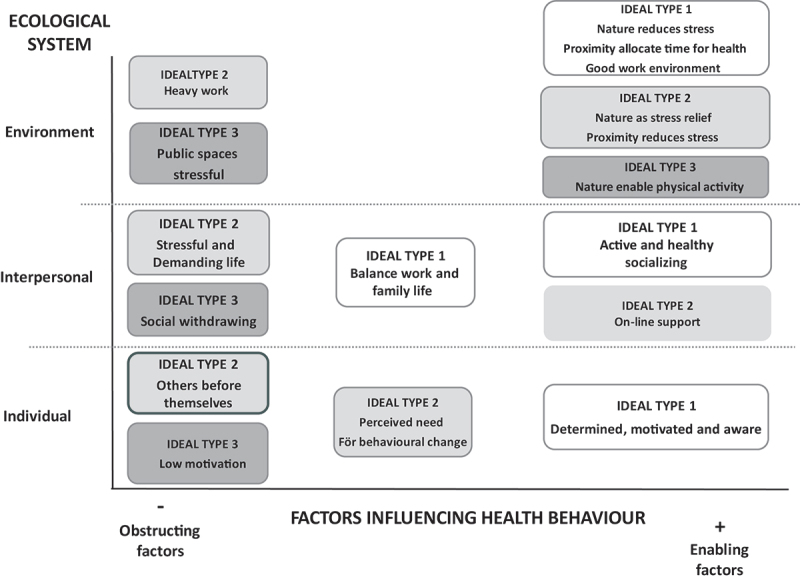
Obstructing and enabling factors in different ecological systems for different ideal types of health identity.

The positional map in Figure 2 illustrates the identified obstructing and enabling factors for health behaviours in different ecological systems for each ideal type.

**On individual system level**, ideal type 2 and 3 face several obstructing factors for health behaviours. For ideal type 2, the tendency to put others before themselves is a considerable obstacle in prioritizing their own health. Even if their perceived need for behavioural change is strong, this not a sufficient enabling factor at the individual level (therefore placed in the middle of the figure), since this is subordinated other’s needs. For ideal type 3, low motivation for any health behavioural change becomes an obstacle for behavioural change at individual level. This is partly because they feel well overall, despite some classical risk factors such as being overweight or sedentary.

On the contrary, ideal type 1 expresses several enabling factors for health behaviours at the individual level, since they manage and force themselves to find opportunities for a healthy lifestyle. This ideal type is determined to live a healthy life, motivated to carry out healthy behaviours and aware of what they need to feel good and avoid future ill health.

**On interpersonal system level**, ideal type 2 faces a stressful and demanding life situation with significant needs among family members, which obstructs them from finding time for physical exercise or preparing healthy meals. For ideal type 3, the tendency of social withdrawing becomes an obstacle for health behaviours, since they e.g., avoid exercising with other people around. Thus, having a “training buddy” does not appeal to them.

For ideal type 1, balancing between work and family life implies challenges that can obstruct health behaviours. Still, this ideal type tries to make conscious choices to balance work and family life to enable time to prioritize their health, such as working part-time and lowering their expectations and demands for housework. Hence, this potential obstacle to healthy behaviour is balanced (and thus placed in the middle of the figure).

In addition, ideal type 1 combines their social life with physical activities, resulting in active and healthy socializing, which become an enabling factor for health behaviours for them at interpersonal system level. For ideal type 3, access to online social support is a factor that can potentially enable health behaviour change. The social support they lack in real life is compensated for by involvement in e.g., social media platforms

**On environmental system level** a heavy work situation is an obstacle to a healthy lifestyle for ideal type 2. Heavy physical work wears on the body and leaves little energy for further activities after work for them. For ideal type 3, an obstacle on the environmental level is the tendency to find public spaces stressful, which makes it difficult to go to e.g., public gyms or other arenas for physical activities.

All three ideal types express enabling factors for health behaviours on environmental system level. Ideal type 1 enjoys spending time in nature, which is described as reducing stress. In addition, proximity to work and school allocates time for health behaviours by allowing daily walking or biking to get there. A good work environment with good social relations and time for exercising during working hours also enables a healthy lifestyle for this ideal type. Likewise, ideal type 2, views spending time in nature as stress relief and, as such, as an enabler for a healthy life. In addition, proximity to work and school reduces stress and facilitates the demanding everyday life for them. Like the other two ideal types, ideal type 3 enjoys being outdoors in nature and views nature as enabling physical activity.

## 5. Discussion

This study illustrates how obstructing and enabling factors for health behaviours differ in patterned ways for people with various health identities. According to Grabowski (2015), health identities—defined as “observations and expectations concerning one’s own health and knowledge about health and in what ways one’s own health is related and comparable to the health of others” (p. 141, slightly revised)—are communicatively constructed and, as such, are flexible and can be affected and changed. This implies that a health identity is not static but could be reconstructed in interaction with the surrounding environment.

Having a health identity in which health is viewed as the main priority in one’s life (ideal type 1) implies high motivation and agency to change and maintain health behaviours. Personal motivation and health literacy interact with the environment to enable a healthy lifestyle for those with this health identity. From a social-ecological and health identity perspective, this could be viewed as a mutual influence between the individual and her environment (Grabowski, 2015; McLeroy et al., 1988). A physically active individual can inspire people in the surrounding environment to become active too, and vice versa; an environment encouraging physical activity makes it easier to retain healthy behaviour (McLeroy et al., 1988; Nieboer & Cramm, 2019).

In contrast, having a health identity that considers that life stands in the way of health (ideal type 2) may involve feeling powerless to change one’s health behaviour. Interactions between a self-image that puts others before oneself and a demanding everyday life constitute significant obstacles to health behaviour changes for people with this type of health identity. From a social-ecological perspective, the mutual influence between this individual and their environment may involve an individual who is sensitive to others’ needs, encouraging the social environment (e.g., a family or a workplace) to expect support, which further strengthens that individual’s perception that other’s needs are more important than their own needs and health. Nieboer and Cramm (2019) investigated environmental factors influencing physical activity among older people in the Netherlands. They found that participants who stated that it was important to them for others to agree with their healthy lifestyle were less physically active than those who responded negatively to the same question. This finding indicates that being attentive to approval from others can become an obstacle to health behaviour change.

Having a health identity that does not view health as a major concern in life (ideal type 3) provides low motivation for a healthy lifestyle. The interaction between low motivation at the individual level and a tendency of social withdrawing at the interpersonal levels and a social environment that expects adherence to health norms becomes an obstacle to health behavioural change among people with this health identity. An introvert with potential previous negative experiences of social networks prefers to withdraw from social situations; moreover, the more one deviates from societal norms about health (i.e., being physically fit), the less likely one is to follow these health norms. As outlined by others, individuals who identify with a social group are more prone to follow that social group’s (health) norms (Dempsey et al., 2018). The ideal type 3 in this current study does not have an evident social identification group; instead, they view themselves as deviating from other people in many ways, becoming an additional obstacle to health behavioural change.

Our results suggest that social-ecological interventions for behavioural change could be more effective if adapted to the specific needs of people with different health identities. In addition to screening for various risk factors, screening for health identities—that is, how individuals view health and their agency to influence their health—could be helpful in the planning and designing of social-ecological health-promoting interventions.

### 5.1. How can tailor-made social-ecological models be created in health-promotion interventions?

Our results suggest that people with a health identity that puts health as the main priority in life (ideal type 1) require little if any, interventions from health services to support health behavioural changes. Others (Pirie et al., 1986) found that people who already adhere to health-promoting behaviours are more prone to participate in health-promotion interventions such as CVD screening. Likewise, people with the least severe mental health problems are more eager to seek help than those with more serious mental health problems (Velasco et al., 2020). In contrast, people with a health identity that considers that life stands in the way of health (ideal type 2) or that health changes are unnecessary or impossible (ideal type 3) might require more support from the health system to manage necessary health behavioural changes.

From a social-ecological perspective, our results illustrate that, for ideal types 2 and 3, the obstacles to health behavioural change exist at the individual, interpersonal, and environmental system levels, although the specific difficulties differ for each ideal type. For ideal type 2, the main obstacle on an individual level relates to the inability to prioritize their needs and set boundaries with others. For ideal type 3, the obstacles on the individual and interpersonal levels involve a lack of motivation and low trust in support from others. These needs go beyond the purely biomedical model (Engel, 1977). However, they could still be met within health services by psychological and social counselling, which are usually available in primary healthcare in Sweden. However, a main obstacle for ideal type 2 is the need to unload their heavy burden on an interpersonal level, which requires balancing responsibilities for family, domestic and paid work. Health services do not traditionally meet these needs, even though almost 50% of all sick leave in Sweden is related to stress or mental health (The Swedish Social Insurance Agency, 2022, 2023). One could argue that health services should acknowledge strategies to meet these significant needs, given the substantial negative impact on individuals and society.

An interesting example of how to intervene on an interpersonal level to influence health behaviour is how private gyms and other sport facilities offer child rooms, babysitting or the option to bring babies to the gym (see e.g., Friskis & Svettis, 2022). This is an illustrative example of how unloading and helping with balancing family and domestic duties could facilitate health behaviour change. Mailey et al. (2014) explored barriers and facilitators for physical activity among working mothers and fathers against the background that parenthood is consistently associated with a decline in physical exercise. Family responsibilities, work, scheduling constraints and lack of support were reported as barriers to physical activity, while support and “pushing” from others helped them become active. In addition, the ability to be active with children and during children’s activities was perceived as a facilitator for physical activity among parents (Mailey et al., 2014). Increasing the collaboration between health services and public, private and civil society actors could benefit the planning and design of interventions at the interpersonal level to support health behavioural change. This also appeals to the enabling factors for health behavioural change in this study. For ideal type 1, active and supportive socializing with others acts as an important enabling factor at the interpersonal level. The potential health-promoting effects of social support and social networks have long been known (Berkman & Glass, 2000). Our results illustrate how the lack of social support for ideal types 2 and 3 obstructs health behavioural change. Examples of interventions to increase social support for health-promoting purposes do exist (Behrendt et al., 2023; Paquet et al., 2023) but are still relatively rare within the health system in the Swedish setting. For example, these interventions involve setting up self-help groups, developing mentor or buddy systems, training network members in providing social support (Heany & Israel, 2002), or carrying out activities to increase social interactions or instrumental support (Paquet et al., 2023). Our results confirm that initiating social supportive networks is a valuable health-promoting strategy that could be further developed within the health system. Interventions to promote online social support might be of particular importance for individuals with a demanding family and work situation that limits time for socializing (particularly visible for ideal type 2 in this study). In line with this, Naslund et al. (2016) argue that online peer-to-peer connections may provide opportunities for individuals with serious mental illness to challenge stigma and get access to online interventions for wellbeing. Our ongoing Star C programme (Ng et al., 2021) aims to develop a technical tool for personalized digital coaching to support health behavioural change. It is reasonable to suppose that this kind of digital tool might benefit people with a health identity like ideal type 2 in this study.

Regarding factors in the environmental system, all ideal types underline the health-promoting effects of spending time in nature. The health-promoting effects of green spaces/nature are well-known in research (Eriksson & Emmelin, 2013; Jimenez et al., 2021; Nguyen et al., 2021). Access to green areas mitigates stress and promotes physical activity (Fan et al., 2011). In their narrative review, Jimenez et al. (2021) found positive associations between nature exposure and many health outcomes, such as improved cognitive function, brain activity, blood pressure, mental health, physical activity and sleep. Examples of interventions that use physical activity as a prescription already exist (The Public Health Agency of Sweden, 2024; Rooney et al., 2023). Our results suggest that providing “time in nature as a prescription” could be a health-promoting intervention. These kinds of interventions would most probably benefit from collaboration between health services and outdoor organizations.

## 6. Conclusions and implications for health promotion and health interventions

This study illustrates how obstructing and enabling factors for health behavioural change exist on the individual, interpersonal and environmental levels. Furthermore, our results indicate that these influencing factors at different ecological system levels interplay in a patterned way depending on health identity—how individuals view their health and their agency to influence their health. Therefore, screening for health identity within the health system might help suggest tailor-made health and behavioural change interventions. Screening for health identities among children and adolescents has been proposed by others (Grabowski, 2015). Still, it could be most useful within population-based health interventions for middle-aged individuals, such as the VIP programme in which our participants were enrolled.

Our results further illustrate how many obstructing and enabling factors identified at different ecological levels refer to needs not usually addressed by the health system. However, since these unmet needs have detrimental effects on health and counteract health behavioural change, there might be a need for the health system to acknowledge factors that go beyond the traditional medical model. This aligns well with the WHO’s SDH approach (WHO Commission on the Social Determinants of Health CSDH, 2008, p. 1), which states,

Maldistribution of health care – not delivering care to those who most need it – is one of the social determinants of health. But the high burden of illness responsible for appalling premature loss of life arises in large part because of the conditions in which people are born, grow, live, work, and age … Action on the social determinants of health must involve the whole of government, civil society and local communities, business, global fora, and international agencies.

Thus, targeted health-promoting interventions require multi-sectorial collaboration between health and other public, private and civil society actors. The WHO Commission for the Social Determinants of Health (WHO CSDH) gathered global examples of how multi-sectorial action on the SDH could be set up. This gathering of examples, in combination with the screening of health identities, could serve as a valuable base for suggesting tailor-made social-ecological health-promoting interventions for behavioural change.

## 7. Methodological considerations

The ability to judge the trustworthiness of a qualitative study largely relies on transparency and reflection about how the sampling, data collection and analysis were designed to address the research questions (Dahlgren et al., 2004). In this study, individual interviews were a suitable data-collection method since we wanted to capture in-depth experiences and perceptions about health and health behavioural change (Charmaz, 2006). Focus group discussions would not have allowed the same range and depth of individual experiences of health and health-related behaviours. Still, they would instead have yielded collective attitudes and beliefs about the same (Wyatt Seal et al., 1998).

The prolonged engagement by three lead researchers permitted the triangulation of investigators and strengthened the study’s trustworthiness (Dahlgren et al., 2004). ME, LS and KL were jointly involved in all stages of the study, from the study’s design to the data collection and analysis, and met regularly for intensive peer-debriefing throughout the study. Their multidisciplinary perspective further strengthened the analysis. ME is a social worker with a PhD in public health, conducting research on the social determinants of health with a particular focus on social networks and social capital. LS is a psychologist with a PhD in public health, conducting research on health behaviour, mental health, and health system. KL is a nutritionist, also with a PhD in public health, and conducting research on complex health interventions. In addition, AS, HL and NN joined the later stages of the data analysis and paper writing, which added further important peer validation of the trustworthiness of the results. Using GT involves going beyond the descriptive level to construct analytical categories that can be useful beyond the specific research situation. Hence, we believe that our constructed ideal types of health identity and identifying obstructing and enabling factors affecting health behavioural change could be transferable to other settings. However, this is for further research to explore and judge.

In our sampling, we aimed to invite participants with various backgrounds and experiences, such as different areas of residence, educational length, sex and age groups. However, our analysis did not identify any significant pattern differences based on these criteria. The relatively small number of participants (typical in qualitative research) and the low proportion of men (only four out of 17 participants) limited the possibility of analysing differences between groups. However, sex and age likely influence people’s health identity and health behavioural change, and further research is needed to explore this. A further step could be constructing instruments to capture different health identities, which could be used in more extensive quantitative surveys.

## Data Availability

The participants of this study did not give written consent for their data to be shared publicly, so due to the sensitive nature of the research supporting data is not available.
